# Fourteen days of smoking cessation improves muscle fatigue resistance and reverses markers of systemic inflammation

**DOI:** 10.1038/s41598-021-91510-x

**Published:** 2021-06-10

**Authors:** Mohammad Z. Darabseh, Thomas M. Maden-Wilkinson, George Welbourne, Rob C. I. Wüst, Nessar Ahmed, Hakima Aushah, James Selfe, Christopher I. Morse, Hans Degens

**Affiliations:** 1 Department of Life Sciences, Centre of Musculoskeletal Sciences and Sport Medicine, Manchester Metropolitan University, John Dalton Building; Chester Street, Manchester, M1 5GD UK; 2 Academy of Sport and Physical Activity, Faculty of Health and Wellbeing, Collegiate Campus, Sheffield Hallam University, Sheffield, UK; 3 Laboratory for Myology, Faculty of Behavioural and Movement Sciences, Amsterdam Movement Sciences, Vrije Universiteit Amsterdam, Amsterdam, The Netherlands; 4 Department of Life Sciences, Centre for Bioscience, Manchester Metropolitan University, John Dalton Building; Chester Street, Manchester, M1 5GD UK; 5 Virology Department, Royal Liverpool and Broadgreen University Hospitals NHS Trust, Liverpool, UK; 6 Department of Health Professions, Faculty of Health, Psychology and Social Care, Manchester Metropolitan University, Brooks Building; 53 Bonsall Street, Manchester, M15 6GX UK; 7 Department of Sport and Exercise Sciences, Centre of Musculoskeletal Sciences and Sport Medicine, Manchester Metropolitan University, All Saints Building; Oxford Street, Manchester, M15 6BH UK; 8 Institute of Sport Science and Innovations, Lithuanian Sports University, Kaunas, Lithuania

**Keywords:** Respiratory tract diseases, Physiology

## Abstract

Cigarette smoking has a negative effect on respiratory and skeletal muscle function and is a risk factor for various chronic diseases. To assess the effects of 14 days of smoking cessation on respiratory and skeletal muscle function, markers of inflammation and oxidative stress in humans. Spirometry, skeletal muscle function, circulating carboxyhaemoglobin levels, advanced glycation end products (AGEs), markers of oxidative stress and serum cytokines were measured in 38 non-smokers, and in 48 cigarette smokers at baseline and after 14 days of smoking cessation. Peak expiratory flow (*p* = 0.004) and forced expiratory volume in 1 s/forced vital capacity (*p* = 0.037) were lower in smokers compared to non-smokers but did not change significantly after smoking cessation. Smoking cessation increased skeletal muscle fatigue resistance (*p* < 0.001). Haemoglobin content, haematocrit, carboxyhaemoglobin, total AGEs, malondialdehyde, TNF-α, IL-2, IL-4, IL-6 and IL-10 (*p* < 0.05) levels were higher, and total antioxidant status (TAS), IL-12p70 and eosinophil numbers were lower (*p* < 0.05) in smokers. IL-4, IL-6, IL-10 and IL-12p70 had returned towards levels seen in non-smokers after 14 days smoking cessation (*p* < 0.05), and IL-2 and TNF-α showed a similar pattern but had not yet fully returned to levels seen in non-smokers. Haemoglobin, haematocrit, eosinophil count, AGEs, MDA and TAS did not significantly change with smoking cessation. Two weeks of smoking cessation was accompanied with an improved muscle fatigue resistance and a reduction in low-grade systemic inflammation in smokers.

## Introduction

Cigarette smoking still is a public health concern and a risk factor for many chronic diseases, including chronic obstructive pulmonary disease (COPD), lung cancer and cardiovascular diseases^1,2^. It is the leading cause of preventable death and 77,900 deaths in the United Kingdom were directly or indirectly attributable to smoking in 2016 ^3^. In England, between 2017 and 2018, an estimated 489,300 smoking-related admissions to hospitals were reported^4^.

The adverse health effects are a consequence of a combination of thousands of toxic and/or carcinogenic substances, including carbon monoxide (CO), reactive glycation compounds, known as glycotoxins, and nicotine in cigarette smoke^5–7^. In addition, the low-grade systemic inflammation and oxidative stress in smokers increases the risk of atherosclerosis^8–12^. Smoking is associated with elevated serum cholesterol and triglyceride levels, impaired glucose tolerance and reduced insulin sensitivity^13^. It has been reported that in diabetic people, a reduced insulin sensitivity could lead to glycation of myofibrillar proteins^14^ that may be further aggravated by glycotoxins in cigarette smoke that can also react with serum proteins to form advanced glycation end products (AGEs)^6^.

In addition to the health burden of cigarette smoking and the potential adverse effect on respiratory function^15,16^, smoking can also have a negative impact on muscle function^17–19^. Part of the potential detrimental effect of cigarette smoking may be attributable to the negative impact on the oxygen delivery to tissues, including skeletal muscles, that may in turn result in exercise intolerance and a reduced muscle fatigue resistance^20–22^. Such an impaired oxygen delivery is at least partly attributable to the CO in the cigarette smoke that strongly binds to haemoglobin (Hb), forming carboxyhaemoglobin (COHb)^23^. This not only reduces the oxygen carrying capacity of the blood, but also causes a left-shift of the Hb-dissociation curve. The significance of elevated COHb levels has been illustrated by an acute CO-induced reduction in muscle fatigue resistance in healthy people^24^. In addition, CO and cyanide may also directly impair mitochondrial respiration^25,26^. As fatigue resistance was similar in COPD patients who had quit smoking and healthy age-matched non-smokers^27^, we hypothesised that the effect of smoking on skeletal muscle fatigue is readily reversible by smoking cessation .

Smoking cessation is an important step to stop or reverse many of the detrimental effects of smoking and is considered a highly effective way to reduce morbidity and mortality^28^ and slow down the accelerated decline in FEV_1_^29,30^. In fact, smoking cessation is considered one of the main actions to attenuate the progression of COPD^31,32^. In line with this, it has been seen in mice, that the smoking-induced lung inflammation, mitochondrial dysfunction, limb and diaphragm muscle atrophy, and elevated IL-1α and TNF-α levels were normalised after smoking cessation^26,33^. In addition, if CO is an important cause of a reduced muscle fatigue resistance and exercise tolerance, we expect that smoking cessation, resulting in a quick normalisation of the COHb levels^34^, will be associated with a concomitant improvement in muscle function. Therefore, we hypothesise, that just two weeks of smoking cessation is sufficient to detect measurable improvements in muscle fatigue resistance, and diminished levels of circulating inflammatory markers and oxidative stress. As there is some indication that smoking may cause a larger reduction in pulmonary function than in men^35^ and that women have a higher muscle fatigue resistance than men^36^ we were also interested in potential sex differences in the response to smoking cessation.

## Methods

### Participants

Cigarette smokers (men n = 28; women n = 20) and non-smokers (men n = 23; women n = 15) were recruited from the local community and Manchester Metropolitan University (MMU). Participants were 18 to 44 years old, and smokers had smoked for ≥ 1 year and ≤ 17 years. All participants self-reported as being free of symptoms of chronic diseases. In cigarette smokers, all measurements were repeated after 14 days of smoking cessation. The study was approved by the Science and Engineering Research Ethics and Governance Committee at MMU (Ethics reference number: 5944) and performed in accordance to the principles stated in the Declaration of Helsinki. All participants provided written informed consent before participating.

Height and body mass were assessed using a stadiometer and digital scales, respectively. Body mass index (BMI) was calculated. Smoking history was assessed by questionnaire. Smoking volume (SV) was given as pack years, calculated as the current number of packs of cigarettes smoked per day times the number of years smoked.

### Outcome measures

#### Carboxyhaemoglobin (COHb)

A hand-held CO meter (Micro Smokerlyzer, Bedfont Scientific Ltd.; Kent, UK) was used to measure the percentage of the haemoglobin (Hb) oxygen binding sites occupied by CO (%COHb). The measurements were performed according to the recommendations of the manufacturer^37^.

#### Spirometry

Spirometry was conducted using a Micro Medical Spiro USB Spirometer and analysed with Spida 5 software (Cardinal Health, UK). Spirometry was completed in accordance with the American Thoracic Society and European Respiratory Society guidelines^38^. Each participant completed a minimum of three successful manoeuvres with at least 1–2 min rest between each manoeuvre while wearing a nose clip. The manoeuvres were rejected if: participants prematurely stopped exhalation, coughed during the first second of exhalation, lips were not fully sealed around the mouthpiece and/or the effort appeared submaximal. The test session was concluded when the largest two FEV_1_ and the largest two FVC were each within 0.15 L of each other in at least 3 manoeuvres^38^. If these criteria were not met, a maximum of eight manoeuvres were repeated until the criteria were met. Parameters assessed included: FEV_1_, FVC, FEV_1_/FVC ratio, Peak Expiratory Flow (PEF), and predicted values. The coefficient of variation (CV) for FEV_1_, FVC and PEF was 2.09%, 2.25% and 2.80%, respectively.

#### Maximal voluntary contraction (MVC)

A dynamometer chair was used to measure the MVC during knee extension. Participants were seated with the hip joint in 90° flexion, knee joint angle at 80° and the pelvis strapped to minimise accessory movements. All the measurements were performed on the right thigh. During the MVC, participants received verbal encouragement and visual feedback of the torque signal^18,19,24,27^. Participants performed the MVC twice with two minutes rest between each contraction to prevent development of fatigue. Knee extensor (KE) torque was recorded with a digital acquisition system (Acknowledge, Biopac Systems, Santa Barbara, CA, USA) at 200 Hz, and the highest value was reported as maximal muscle strength^18^. The CV for the MVC was 4.24%.

### Voluntary activation (VA) and muscle fatigue resistance

To assess the VA and muscle fatigue resistance of the quadriceps muscle, carbon–rubber pads (7.5 cm × 13 cm, Axelgaard, USA) were used to apply percutaneous electrical stimulation (square wave, pulse width 200 μs; DSV Digitimer Stimulator, Digitimer Ltd., Herts, UK). The cathode was placed over the distal third of the thigh and the anode over the proximal part of the quadriceps. The electrical stimulation voltage was set at 400 V. To assess the supra-maximal current, single pulses were administered at 30-s intervals with increases in current of 50–100 mA to the relaxed muscle until no further increase in torque was noticed.

To assess the VA during an MVC, the interpolated twitch technique was used^18,19^ and calculated as:

$${\text{VA}}\left( \% \right) = \left( {1 - \left( {{\text{superimposed}}\,{\text{twitch}}\,{\text{torque}}/{\text{resting}}\,{\text{twitch}}\,{\text{torque}}} \right)} \right)\,*\,100$$

and had a CV of 5.96%.

The fatigue resistance of the quadriceps muscles was determined by a series of electrically-evoked isometric 30-Hz trains, 1 s on 1 s off, for 2 min at a current that elicited 30% MVC at the start of the test^19,27^. The fatigue index (FI) was calculated as the final torque as a percentage of the initial torque during the series of the isometric contractions. The CV was 6.44%.

### Haematology parameters and oxidative stress biomarkers

From 9 non-smokers and 20 smokers venous blood was collected from the antecubital vein and repeated after 2 weeks of smoking cessation from smokers only. After determination of the haematocrit (%Hct) the blood was collected in 4-mL vacutainers without anticoagulants (BD Vacutainer, Becton Dickinson Company, USA). The blood samples were allowed to clot for 15 min and serum was separated from whole blood by centrifugation (20 min; 500 × g) at room temperature. Following centrifugation, the serum was aliquoted in 1-mL microcentrifuge tubes, frozen and stored at − 80 °C until further analysis.

Serum protein, albumin and glucose concentrations were measured colourimetrically using Biuret reagent Randox kits using RandoxRX Daytona analyser (Randox Laboratories Ltd., Belfast, Ireland). The glucose concentration was determined after enzymatic oxidation in the presence of glucose oxidase. The Hb concentration was determined with a HemoCue (HemoCue® Hb 201 + System). Blood cell counts included agranulocytes (lymphocytes, monocytes) and granulocytes (neutrophils, eosinophils and basophils). Serum cytokines levels were quantified using flow cytometry. Briefly, positive and negative controls were used to set up the flow cytometer (BD FACScalibur, Becton Dickinson Company, USA) and analysed using the high flow setting (FL2 channel), using Cell Quest Pro software and flowcytomix software. The software translated the flow cytometric results into cytokine concentrations (pg mL^−1^). Serum malondialdehyde (MDA), a marker of lipid peroxidation, was quantified spectrophotometrically using a lipid peroxidation kit (Oxford Biomedical Research, UK). The serum total antioxidant status (TAS) in was analysed using the TAS kit (Randox Laboratories Ltd., Belfast, Ireland) according to the recommendations by the manufacturer. The abundance of low molecular weight (LMW) AGEs were assessed using a spectrofluorimeter (BioTek, USA), and total AGEs were assessed by ELISA (Cell Biolabs, United States). All tests were carried out in duplicate and averaged.

### Statistical analysis

Statistical analyses were performed using SPSS 24.0 (IBM Corporation, NY, US). Data were assessed for normality with the Shapiro–Wilk tests. If the data were not normally distributed, non-parametric Kruskal–Wallis H test was performed. A two-way univariate ANOVA with as between factors group (smokers, non-smokers and smoking cessation) and sex was used. If a significant group effect, or a group * sex interaction was found, a Dunnet post-hoc test with as standard group the smokers was performed to locate the significant differences. For the blood parameters, comparisons between smokers and non-smokers, and comparison of smokers before and after cessation were performed with unpaired student t-tests. Differences were considered significant at *p* < 0.05. All data are presented as mean ± SD.

## Results

Anthropometric details of the participants are presented in Table 1. The smoking women in our study had smoked longer and had smoked more pack years than the smoking men (*p* < 0.05; Table 1). For none of the parameters group * sex interactions were found, indicating that there were no significant differences in the responses to smoking and smoking cessation between men and women.

**Table 1:** Demographic data.

	Cigarette smokers		Non-smokers	
	Men (n = 28)	Women (n = 20)	Men (n = 23)	Women (n = 15)
Age (years)	25.4 ± 6.0	31.0 ± 12.8*	26.3 ± 10.5	24.8 ± 7.4
Height (m)	1.78 ± 0.09	1.65 ± 0.09*	1.79 ± 0.07	1.65 ± 0.06*
Mass (kg)	77.5 ± 17.7	67.9 ± 11.4*	77.3 ± 11.6	68.2 ± 14.2*
BMI (kg/m^2^)	24.2 ± 4.5	25.1 ± 4.0	24.1 ± 3.1	24.8 ± 4.3
Smoking duration (years)	7.4 ± 4.3	13.7 ± 12.2*	–	–
Cigarettes per day	12.3 ± 6.0	12.7 ± 5.7	–	–
Smokers pack-years	4.6 ± 2.9	9.6 ± 11.0*	–	–

All data are presented as mean ± SD; BMI: body mass index; *: significantly different from men at *p* < 0.05.

The total protein, albumin and glucose concentrations did not differ significantly between smokers and non-smokers (Table 2). Smokers had higher levels of COHb than non-smokers (*p* < 0.001) and the COHb levels had returned to levels similar to that in non-smokers after 14 days of smoking cessation (Table 3).

**Table 2:** Smoking or smoking cessation did not alter total protein, albumin and glucose serum concentration.

Serum concentrations	Non-smokers n = 9	Smokers n = 20	Stop smoking n = 20	Statistical evaluation (p-value)		
				S versus C	S versus SC	SC versus C
Total protein (g/L)	65.4 ± 2.3	66.0 ± 3.5	64.5 ± 2.7	NS	NS	NS
Albumin (g/L)	43.4 ± 2.8	45.7 ± 2.4	44.7 ± 2.3	NS	NS	NS
Glucose (mmol/L)	5.14 ± 0.75	5.10 ± 0.34	5.33 ± 1.03	NS	NS	NS

All data are presented as mean ± SD; C: Non-smokers; S: smokers; SC: 14 days smoking cessation; NS: not significant.

**Table 3:** The impact of smoking and smoking cessation on white blood cell counts, haematocrit, haemoglobin and carboxyhaemoglobin.

Parameters	Non-smokers n = 9	Smokers n = 20	Stop smoking n = 20	Statistical evaluation (*p*-value)		
				S versus C	S versus SC	SC versus C
WBC (10^9^/L)	6.70 ± 2.16	6.98 ± 1.97	6.89 ± 1.70	NS	NS	NS
**Granulocytes**						
Neutrophil (%)	54.6 ± 4.80	60.3 ± 12.1	58.9 ± 8.50	NS	NS	NS
Eosinophil (%)	3.44 ± 0.87	2.24 ± 1.03	2.25 ± 0.98	< 0.05	NS	< 0.05
Basophil (%)	1.67 ± 0.87	1.17 ± 0.94	1.42 ± 0.85	NS	NS	NS
**Agranulocytes**						
Lymphocyte (%)	34.3 ± 3.60	30.5 ± 11.18	32.2 ± 8.05	NS	NS	NS
Monocyte (%)	6.0 ± 1.23	5.58 ± 1.39	5.58 ± 1.30	NS	NS	NS
**Haematocrit, haemoglobin and carboxyhaemoglobin**						
Hct (%)	41.0 ± 4.6	46.5 ± 2.6	45.6 ± 2.6	< 0.001	NS	< 0.01
Haemoglobin (g/dL)	13.7 ± 1.80	15.5 ± 0.89	15.3 ± 0.89	< 0.001	NS	< 0.01
COHb (%)	0.07 ± 0.03	2.26 ± 1.08	0.1 ± 0.28	< 0.001	< 0.001	NS

All data are presented as mean ± SD; WBC: White blood cells; Hct: Haematocrit; COHb: Carboxyhaemoglobin; C: Non-smokers; S: smokers; SC: 14 days smoking cessation; NS: not significant.

### Spirometry

**Figure 1:**
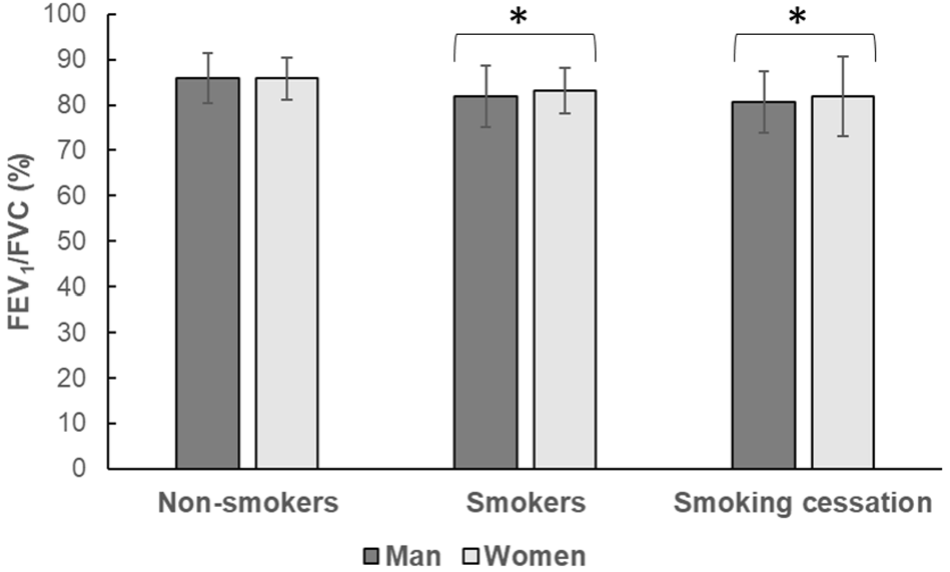
The effect of smoking and 14 days smoking cessation on FEV_1_/FVC: Forced expiratory volume in one second/forced vital capacity; data are mean ± SD; *: significantly different from Non-smokers at p < 0.05.

PEF, FEV_1_ and FVC were higher in men than women (*p* < 0.001), but FEV_1_/FVC, FEV_1pred%_, FVC_pred%_ and PEF_pred%_ did not differ significantly between men and women (Table 4). There was no significant difference in FEV_1_, FEV_1pred%_, FVC_pred%_ and PEF_pred%_ between smokers and non-smokers (Table 4), but PEF (*p* = 0.004; Table 4) and FEV_1_/FVC (*p* = 0.037; Fig. 1) were lower in smokers than in non-smokers. Neither changed significantly over the 14 days of smoking cessation (*p* > 0.05; Fig. 1 and Table 4).

**Table 4:** The effect of smoking and smoking cessation on spirometry, maximal isometric voluntary knee extension torque (KE MVC) and voluntary activation (VA).

Group	Sex	FEV_1_ (L)	FEV_1pred_ (%)	FVC (L)	FVC_pred_ (%)	PEF (L/s)	PEF_pred_ (%)	KE MVC (Nm)	VA (%)
Non-smokers	M	4.4 ± 0.7 (19)	95.9 ± 8.9 (19)	5.20 ± 0.85 (19)	93.4 ± 8.9 (19)	9.90 ± 1.5 (19)	97.9 ± 13.4 (19)	257 ± 66 (14)	94.1 ± 6.8 (14)
Non-smokers	W	3.3 ± 0.4 (14)	98.1 ± 10.9 (14)	3.89 ± 0.50 (14)	98.1 ± 9.6 (14)	7.3 ± 1.2 (14)	98.6 ± 12.5 (14)	175 ± 24 (8)	92.7 ± 13.1 (8)
Smokers	M	4.2 ± 0.7 (21)	92.7 ± 11.8 (21)	5.09 ± 0.8 (21)	93.6 ± 10.1 (21)	9.00 ± 1.5 (21)	90.0 ± 14.1 (21)	238 ± 62 (21)	85.3 ± 14.2 (20)
Stop smoking		4.1 ± 0.8 (7)	90.0 ± 15.7 (7)	5.03 ± 1.0 (7)	90.3 ± 13.1 (7)	9.80 ± 1.9 (7)	95.6 ± 19.8 (7)	270 ± 51 (13)	95.4 ± 4.9 (13)
Smokers	W	3.0 ± 0.419)	92.0 ± 11.0 (19)	3.61 ± 0.4 (19)	93.2 ± 9.2 (19)	6.30 ± 1.2 (19)	89.6 ± 16.0 (19)	149 ± 37 (13)	94.8 ± 7.3 (13)
Stop smoking		2.9 ± 0.5 (8)	91.4 ± 12.6 (8)	3.49 ± 0.4 (8)	91.6 ± 12.6 (8)	6.50 ± 1.1 (8)	90.1 ± 18.2 (8)	150 ± 33 (9)	95.8 ± 4.9 (9)
**P-value**									
Smoke		NS	NS	NS	NS	**0.004**	NS	NS	NS
Sex		** < 0.001**	NS	** < 0.001**	NS	** < 0.001**	NS	** < 0.001**	NS

All data are presented as mean ± SD; FEV_1_: Forced expiratory volume in one second; FVC: Forced vital capacity; PEF: Peak expiratory flow; PEF _pred_ (%): PEF predicted percentage; (x) denotes number of participants. Significant effects are denoted in bold; NS: not significant.

### Muscle function

**Figure 2:**
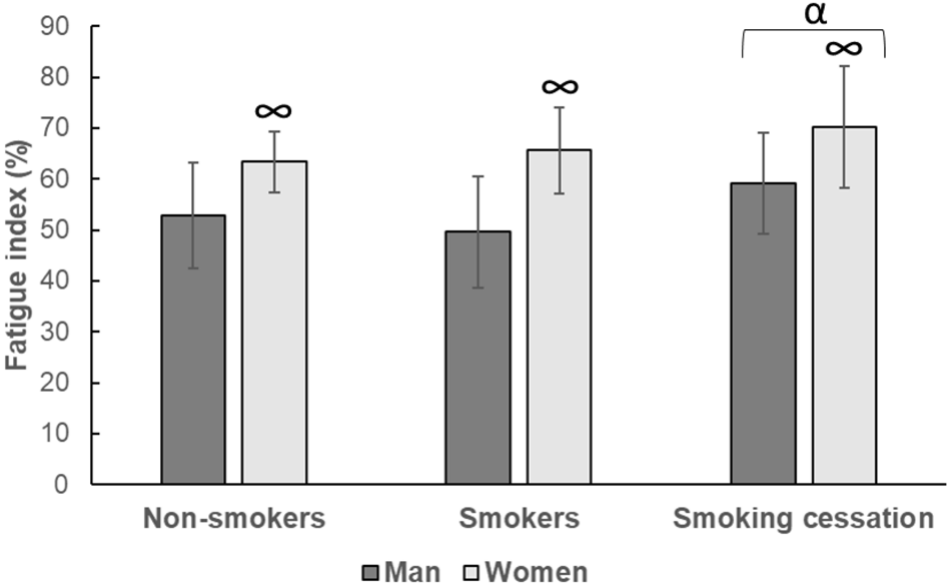
The effect of smoking and 14 days smoking cessation on fatigue index. Data are mean ± SD; ∞: significantly different from men at *p* < 0.05; α: significantly different from smokers at *p* < 0.05.

Knee extension MVC was higher in men than women (*p* < 0.001; Table 4) and FI was higher in women than men (*p* < 0.001), but there were no significant sex differences in VA (*p* = 0.096; Table 4). There was no significant difference in MVC and VA between smokers and non-smokers (Table 4). While there was no significant difference in FI between smokers and non-smokers, smoking cessation resulted in an increased FI (*p* < 0.001; Fig. 2).

### Haematology

There were no significant differences in total white blood cell, neutrophil, lymphocyte, monocyte and basophil counts between smokers and non-smokers (Table 3). The eosinophil count was lower in smokers than non-smokers (*p* < 0.05) even after 14 days smoking cessation (Table 3). Smokers had a higher haemoglobin concentration and haematocrit than non-smokers (*p* < 0.001) and was not changed significantly after 14 days of smoking cessation (Table 3).

### Circulating markers of oxidative stress

**Figure 3:**
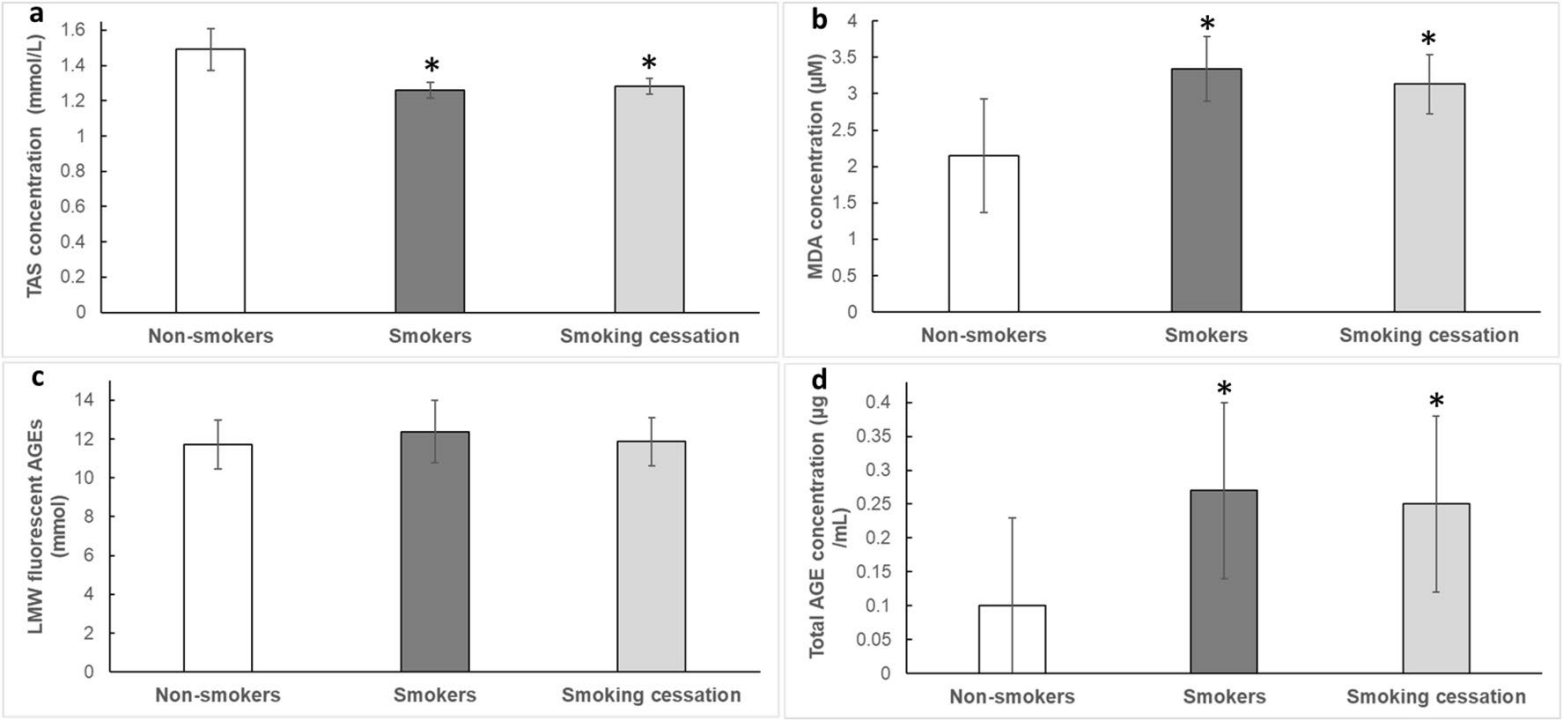
Effects of smoking and 14 days smoking cessation. (**a**) Total antioxidant status (TAS); (**b**) Malondialdehyde concentration; (**c**) Low molecular weight (LMW) advanced glycation end products (AGEs) fluorescence; (**d**) AGEs concentration; data are mean ± SD; *: significantly different from Non-smokers at *p* < 0.05.

The total antioxidant status was lower in smokers than non-smokers (*p* < 0.001) and was not significantly changed after 14 days of smoking cessation (Fig. 3a). Lipid peroxidation, in the form of the concentration of MDA was higher in smokers compared to non-smokers (*p* < 0.001) and were not significantly changed after 14 days of smoking cessation (Fig. 3b). Although the low molecular weight AGE levels did not differ significantly between smokers and non-smokers (Fig. 3c), the total AGE levels were higher in smokers compared to non-smokers (*p* < 0.05; Fig. 3d). Smoking cessation did not have a significantly alter the concentration of AGEs (Fig. 3c,d).

### Circulating levels of cytokines

**Figure 4:**
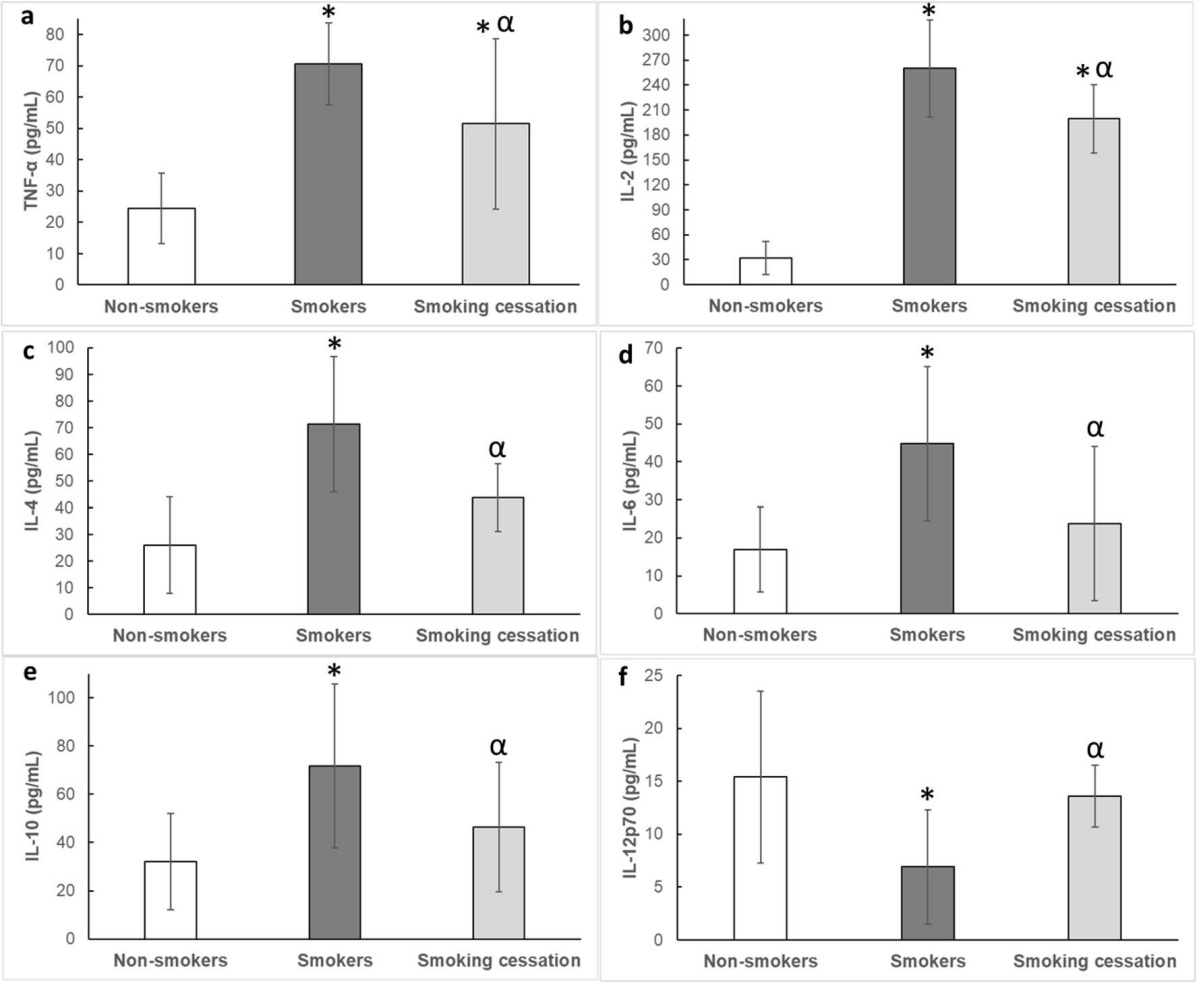
Effects of smoking and 14 days smoking cessation (**a**) TNF-α: tumour necrosis factor-alpha; (**b**) IL-2: interleukin-2; (**c**) IL-4: interleukin-4; (**d**) IL-6: interleukin-6; (**e**) IL-10: Interleukin-10; (**f**) IL-12p70: interleukin-12p70; data are presented as mean ± SD; *: significantly different from Non-smokers at *p* < 0.05; α: significantly different from smokers at *p* < 0.05.

Smokers had higher circulating levels of TNF-α, IL-2, IL-4, IL-6 and IL-10 levels than non-smokers (All *p* < 0.001; Fig. 4a–e), while IL-12p70 levels were lower in smokers than in non-smokers (*p* < 0.001; Fig. 4f). Almost all circulating cytokines concentrations returned to levels seen in non-smokers after 14 days of smoking cessation, except for TNF-α and IL-2 that though reduced, where still elevated in comparison to non-smokers (*p* < 0.05; Fig. 4a,b). TNF-β, IFN-γ, IL-1β, IL-5 did not differ significantly between smokers and non-smokers (Table 5).

**Table 5:** The impact of smoking and smoking cessation on circulating cytokines.

Cytokines (pg/mL)	Non-smokers n = 9	Smokers n = 20	Stop smoking n = 20	Statistical evaluation (p-value)		
				S versus C	S versus SC	SC versus C
TNF-β	82.9 ± 31.2	109.0 ± 34.0	109.1 ± 46.8	NS	NS	NS
IFN-γ	139 ± 82	144 ± 57	161 ± 55	NS	NS	NS
IL-1β	100.4 ± 53.0	109.4 ± 38.4	94.1 ± 41.3	NS	NS	NS
IL-5	109 ± 89	125 ± 57	132 ± 30	NS	NS	NS

All data are presented as mean ± SD; TNF-β: tumour necrosis factor-beta; INF-γ: interferon-gamma; IL-1β: interlukin-1beta; IL-5: interleukin-5; C: Non-smokers; S: smokers; SC: 14 days smoking cessation; NS: not significant.

## Discussion

The main observation of the present study was that in smokers with normal spirometry 14 days of smoking cessation resulted in a normalisation of skeletal muscle fatigue resistance and a return of circulating markers of inflammation. This indicates that even as little as 14 days of smoking cessation can confer measurable benefits that may encourage smokers in their smoking cessation efforts.

### Differences between smokers and non-smokers

#### Spirometry

The present study confirms that FEV_1_, FVC and PEF were higher in men than women^39^. The spirometry in smokers was similar to that of non-smokers, except for a lower FEV_1_/FVC, indicative of some minor developing airway obstruction.

#### Muscle function

In line with previous observations^18,19,40^, we found that the maximal strength of the knee extensor muscles in smokers was similar to that in non-smokers. Others, however, have reported a lower force generating capacity in smokers^41–44^. Although part of a lower strength may at least in theory be attributable to a lower ability to voluntarily activate the muscle, we found no difference in voluntary activation between smokers and non-smokers, and if anything, even an increased VA has been reported in smokers^19^. The latter may be the result of an increased sympathetic nerve activity in smokers, possibly due to a central stimulant action of nicotine^45,46^. Whatever the cause of the discrepancy between studies concerning the impact of smoking on the MVC, it indicates that smoking per se is not necessarily associated with muscle weakness.

Somewhat unexpected was the absence of lower fatigue resistance in smokers that was seen in previous studies using the same fatigue protocol^18,19^. This reduced fatigue resistance in the previous smokers was thought to be at least partly attributable to elevated COHb levels, seen also in the current and other studies^23,47^ that not only reduces the oxygen carrying capacity, but also the release of oxygen due to the left-shift of the Hb-dissociation curve^17,24^. It should be noted, however, that 6% COHb reduced skeletal muscle fatigue resistance^24^ and the 3% COHb in our participants may not have had a measurable impact on the oxygen delivery to the skeletal muscle, and hence the fatigue resistance.

### Blood parameters

While we did not see a significant difference in the albumin and total protein concentrations in the blood of smokers and non-smokers, others did see that smokers had a lower total protein and albumin concentration compared to non-smokers^48^ or even a higher protein concentration^49^. Consistent with previous studies^50–52^, the haemoglobin concentration and haematocrit were higher in smokers compared to non-smokers. The higher haemoglobin concentration may well be an adaptation to maintain the oxygen carrying capacity in the face of elevated COHb levels^53,54^.

Although there were no significant differences in monocytes and lymphocytes between smokers and non-smokers in the current and previous studies^52,55^, except for a reduction in the number of eosinophils, we observed a significant increase in TNF-α, IL-2, IL-4, IL-6 and IL-10. This suggests that smoking activates mononuclear cells to release cytokines. In line with this, it has been observed that cigarette smoke induces the release of TNF-α in an in vitro macrophage model system^56^, but others found no increased release of TNF-α peripheral blood mononuclear cells to cigarette smoke extracts^57^. It should be noted, however, that TNF-α is not only produced by blood mononuclear cells, but also by epithelial cells, fibroblasts and smooth muscle cells^58^, p. 229 and perhaps mononuclear and epithelial cells in the lung of smokers^59^. In line with this, it has been observed that there was a significantly elevated number of macrophages and neutrophils in the broncheo-alveolar lavage fluid of smoking mice^26^. Therefore, lung-derived cytokines may well be the prime explanation of the higher TNF-α, IL-2, IL-4, IL-6 and IL-10 concentrations and the lower level of the anti-inflammatory IL-12p70 concentration in smokers than non-smokers, indicating that even young-adult asymptomatic smokers suffer from a low-grade systemic inflammation.

It is possible that the lower TAS and higher MDA levels in smokers, also reported by others^60^, may be due to this low-grade systemic inflammation. The oxidative stress in smokers may well have contributed to their elevated AGE levels^6,61,62^. Although AGEs are often considered to represent indirectly a high level of glucose^63,64^, we and others^49^ did not find elevated glucose levels in smokers. It should be noted that not only high glucose concentrations, but also toxic constituents of cigarette smoke might induce glycotoxins that rapidly react with protein to form AGE^6^. Therefore, we suggest that the increased AGEs in asymptomatic young-adult smokers is primarily attributable to glycotoxins, oxidative stress, and to some extent secondary to the low-grade systemic inflammation.

### Smoking cessation

#### Spirometry

The present study showed that 14 days of smoking cessation did not result in an improvement in the smoking-induced decrement of FEV_1_/FVC. This is supported by numerous studies suggesting that pulmonary changes induced by smoking are irreversible, even though smoking cessation is the best approach to stop the accelerated decline in lung function in smokers^65–70^.

### Muscle function

In support of our hypothesis, we found an improved skeletal muscle fatigue resistance after 14 days of smoking cessation that was accompanied with a return of the COHb levels to that seen in non-smokers. It therefore does appear that the improved fatigue resistance after smoking cessation was at least to some extent attributable to an improved oxygen delivery, and perhaps also improved mitochondrial function. Indeed, 2 weeks smoking cessation has been shown to improve mitochondrial function in mouse muscle, although in mice this was not accompanied by an improved muscle fatigue resistance^26^. Nevertheless, our data suggest that even in smokers with only 3% COHb smoking cessation can still enhance muscle fatigue resistance, even when the fatigue resistance was not significantly less than that in non-smokers. Perhaps the enhanced fatigue resistance after smoking cessation is to some extent also attributable to the elevated haemoglobin concentration and haematocrit that enhance the oxygen carrying capacity and oxygen delivery with smoking cessation even above that seen in the non-smokers^17,53^, similar to that seen after doping with erythropoietin^71^. In addition, smoking cessation also improves exercise-induced vasodilation^72,73^. Overall, our data indicate that even smoking cessation for as short a period as 2 weeks can result in measurable improvements in muscle fatigue resistance.

### Blood parameters

Another significant observation in our smokers was evidence of low-grade systemic inflammation and oxidative stress. It was therefore particularly interesting to assess the impact of smoking cessation on these parameters. Here we found that most of the abnormal blood parameters were normalised by 14 days of smoking cessation.

The present study showed that both TAS and MDA did not return to normal levels after 14 days of smoking cessation. This may occur later as it has been shown that after 28 days of smoking cessation, TAS was increased and MDA levels reduced back to normal levels^74^. AGE levels also did not show a significant decrement after 14 days of smoking cessation. The 3-week half-life of AGEs^75–77^ may explain that despite the diminished low-grade systemic inflammation AGEs remained elevated. Therefore, 14 days of smoking cessation might not be long enough to cause a normalisation in TAS, MDA and AGEs to levels similar to that in non-smokers.

Smoking cessation interrupts the exposure to chemicals in cigarette smoke ^28^ and it is likely that the reduced concentration of smoking-related chemicals in the blood that induce inflammation will result in a reduction in cytokine levels^78–80^. For example, the elevated levels of TNF-α after 20 weeks smoking was back to baseline levels after 8 weeks smoking cessation in the broncho-alveolar lavage fluid of mice^33^ and similarly 30 days smoking cessation resulted in a significant reduction in TNF-α in humans^28^. Here we showed that IL-6, IL-10, IL-12p70, IL-4 returned to normal levels and TNF-α was reduced after 14 days of smoking cessation. It has been suggested that the lungs are the primary cause of the low-grade systemic inflammation in patients with chronic obstructive pulmonary disease^81^. In line with this it has been shown that 2 weeks smoking cessation in mice led to a return in the number of leucocytes in the broncheo-alveolar lavage fluid to normal levels^26^. Eosinophil numbers remained lower in smokers than non-smokers after 14 days of smoking cessation, which may be secondary to the higher concentration of IL-2 in smokers, even after 14 days of smoking cessation.

### Future directions

We showed significant improvements in muscle fatigue resistance and inflammatory status that may well be sufficient to stimulate smokers in their attempts to quit smoking. Future studies are recommended to conduct longer duration of smoking cessation programmes with larger sample size to assess whether also the markers of oxidative stress and circulating AGEs return to normal values. Although it remains to be seen to what extent the effects observed are related to the duration of smoking, in our previous work we have shown that at least the lower fatigue resistance in smokers was not related to the duration of smoking or smoking pack years^19^.

## Conclusion

Even in smokers with normal spirometry there is significant evidence of oxidative stress and systemic inflammation. A short period of smoking cessation of just 2 weeks is enough to improve the inflammatory status to almost back to normal levels and induce an improvement in muscle fatigue resistance. These benefits will undoubtedly stop the progression of detrimental effects of low-grade systemic inflammation and encourage smokers in their attempts to quit smoking.

## Data Availability

When appropriate and reasonable, anonymised data are available upon request from the corresponding author.
